# The Future Relations of Medicine and the State

**Published:** 1914-05-23

**Authors:** 


### THE FUTURE RELATIONS OF MEDICINE AND THE STATE.

In another column (p. 223) will be found some very timely warnings addressed recently by Dr. Macnaughton Jones to a Hampstead medical audience concerning the possible and probable results of recent legislation upon the future of medicine and of its relations with the State. These warnings apply with especial force, naturally, to the medical profession; but institutional workers, and even the commonwealth at large, may do worse than ponder them carefully, for there is much in his essay which it behoves all good citizens to reflect upon. His main indictment of the present-day attitude of the State, or rather of Parliament, towards medicine is that far-reaching measures are adopted and passed into law without sufficient previous detailed consideration of their effects, immediate or remote. That this complaint is well founded can hardly, we suppose, be denied, and it is a very serious reproach to statesmanship that such a charge should be even conceivable, let alone incontestable. Admittedly, the process of forecasting all, or even a proportion, of the future results of any particular law is exceedingly difficult, if not impossible; but the gravamen of Dr. Macnaughton Jones' charge is that no real attempt is made to perpend even the most obvious and momentous consequences to be expected when grandiose but Utopian schemes of national health are set on foot by politicians who in medical respects are distinctly amateur reformers.

The fact is, as we conceive, that the national consciousness of the importance of hygiene and preventive medicine, as well as of the vast strides in actual therapeutics, has so far developed that party wire-pullers see their chance of exploiting it. It appears not to be worth anybody's while, as yet, to build on surer foundations than the quicksands of party politics, or to plan boldly for the future on sound principles. A jerry-built ramshackle structure which will keep out the rain for a year or so seems to be the highest achievement that anyone can rise to. And to patch it as often as it springs a leak is thought a sufficient answer to expert criticisms of the architecture.

Sooner or later, of course, the fruits of this policy will ripen; and it will be evident to all that to tinker piecemeal with the vast problems of the health of the people is unsound. When that day comes the institutional world, as well as the medical profession, must be prepared to play a part in the constructive statesmanship which will be required. On the last occasion, some three years ago, when this need arose, neither the medical profession nor the institutional world knew its own mind or spoke with certain voice. In these columns the emergency was at once appreciated, and a coherent line of policy expounded, not altogether without avail. But, broadly speaking, the importance of the crisis was not grasped, and opportunities were missed which may not occur in so favourable a form.

So far, the Insurance Acts have not given a death-blow—as many people feared would be the case—to the voluntary system in this country. It is true that a small number of comparatively unimportant institutions have had to close; and it is also true that a great majority of the hospitals have found greater difficulty in discharging their responsibilities. Whether this state of things will continue we are not bold enough to foretell. The trend of twentieth-century finance, as exemplified in the 1914 Budget, is very significant. It aims, quite clearly, at a more uniform distribution of wealth than now exists. This may be a desirable end in itself, but one of its corollaries would seem likely to be a lessening in the volume of private charity, whether in the form of subscriptions or legacies; and in such a change the voluntary hospitals will suffer severely. Here, then, is an instance of an omission such as Dr. Macnaughton Jones complains of: the omission, that is, to look ahead and anticipate the probable results of present-day policies. Quite imaginably the able authors of these policies do foresee, and indeed desire, the ultimate effects of their actions. But most certainly many of those who support them do not do anything of the sort, and will be unpleasantly surprised when the inevitable consequences eventuate. A State Medical Service, as is well known, commends itself to certain ardent reformers. If voluntary effort fails to keep the hospitals going, we know of nothing so likely to lead to the nationalisation of medicine as the State control of institutions which would follow. To drift into this for want of a little clear thinking is not worthy of a learned profession; and if Dr. Macnaughton Jones' words lead to more of that difficult mental exercise, he will have rendered a great service not only to his colleagues, but to the whole community.

